# Comprehensive assessment of regulatory T-cells-related scoring system for predicting the prognosis, immune microenvironment and therapeutic response in hepatocellular carcinoma

**DOI:** 10.18632/aging.205649

**Published:** 2024-03-08

**Authors:** Bitao Jiang, Xiaojuan Ye, Wenjuan Wang, Jiajia He, Shuyan Zhang, Song Zhang, Lingling Bao, Xin Xu

**Affiliations:** 1 Department of Hematology and Oncology, Beilun District People’s Hospital, Ningbo, China; 2 Radiotherapy Department, The Second People’s Hospital of Wuhu, Wuhu, China; 3 Department of Hematology and Oncology, Ningbo Yinzhou No. 2 Hospital, Ningbo, China; 4 Pharmacy Department, Beilun District People’s Hospital, Ningbo, China

**Keywords:** regulatory T cell, tumor immune microenvironment, prognosis, biomarker, hepatocellular carcinoma

## Abstract

Introduction: Regulatory T cells (Tregs) play important roles in tumor immunosuppression and immune escape. The aim of the present study was to construct a novel Tregs-associated biomarker for the prediction of tumour immune microenvironment (TIME), clinical outcomes, and individualised treatment in hepatocellular carcinoma (HCC).

Methods: Single-cell sequencing data were obtained from the three independent cohorts. Cox and LASSO regression were utilised to develop the Tregs Related Scoring System (TRSSys). GSE140520, ICGC-LIRI and CHCC cohorts were used for the validation of TRSSys. Kaplan-Meier, ROC, and Cox regression were utilised for the evaluation of TRSSys. The ESTIMATE, TIMER 2.0, and ssGSEA algorithm were utilised to determine the value of TRSSys in predicting the TIME. GSVA, GO, KEGG, and TMB analyses were used for mechanistic exploration. Finally, the value of TRSSys in predicting drug sensitivity was evaluated based on the oncoPredict algorithm.

Results: Comprehensive validation showed that TRSSys had good prognostic predictive efficacy and applicability. Additionally, ssGSEA, TIMER and ESTIMATE algorithm suggested that TRSSys could help to distinguish different TIME subtypes and determine the beneficiary population of immunotherapy. Finally, the oncoPredict algorithm suggests that TRSSys provides a basis for individualised treatment.

Conclusions: TRSSys constructed in the current study is a novel HCC prognostic prediction biomarker with good predictive efficacy and stability. Additionally, risk stratification based on TRSSys can help to identify the TIME landscape subtypes and provide a basis for individualized treatment options.

## INTRODUCTION

The latest global statistical survey on 36 cancers in 185 countries revealed that the liver cancer is the third most common malignant tumour with the highest mortality rate worldwide [1]. Among them, hepatocellular carcinoma (HCC) is the most prevalent type of liver cancer, accounting for about 75% to 85% of liver cancer cases [2, 3]. Radical surgery is the optimal treatment for early-stage HCC; however, postoperative metastases occur frequently and affect patient prognosis [4]. Furthermore, most individuals with HCC are usually diagnosed at an advanced stage due to the lack of effective biomarkers and obvious early symptoms [5]. With the exploration of the molecular mechanism of HCC and the development and use of targeted drugs in recent decades, the situation of patients has improved, but the 5-year survival rate is still not optimistic [6]. Additionally, the remarkable heterogeneity of HCC greatly affects the clinical outcomes, as well as the clinical efficacy of antitumor drugs [7, 8]. Therefore, exploring prognostically relevant molecular biomarkers in patients with HCC is essential to improve the quality of life and enhance the efficacy of antitumor therapy [9].

Tumour microenvironment (TME) is a system in which cancer and non-cancer cells come together and interact [10]. In addition to cancer cells, various immune cells and stromal cells and their released substances are also abundant in the TME and control the TME’s immune status to influence the immunotherapy efficacy. Among them, regulatory T cells (Tregs), as important immunosuppressive regulatory cells, play an important role in tumor immunosuppression and immune escape [11, 12], and are closely related to the prognosis of individuals with tumour [13, 14]. Additionally, tumor-infiltrating Tregs in tumor immune microenvironment (TIME) are also considered as potential targets for immunotherapy and may be used as monotherapy and/or in combination with immune checkpoint blockers (ICBs) [15, 16]. Furthermore, blocking the binding of the immune checkpoints PD-L1/PD-1 selectively interferes with the inhibitory effects of Tregs on T effector cells in HCC patients, thereby suppressing tumor activity [17]. Therefore, exploring biomarkers associated with Tregs can better understand the role of Tregs in HCC and provide a basis for prognostic assessment and selection of individualized treatment options for HCC.

In this study, a Tregs-related scoring system (TRSSys) was developed. Comprehensive validation and evaluation of the system confirmed that TRSSys has high stability and adaptability and is an excellent biomarker for predicting clinical outcomes in individuals with HCC. Furthermore, population stratification based on TRSSys can identify HCC patients with different immune landscapes, determine the immune cell infiltration status of different populations, and thus relatively differentiate between immune “hot tumors” and “cold tumors”. Furthermore, TRSSys also helps to determine the relative advantageous population for immunotherapy, which provides a basis for the scheduling of individualized treatment regimen.

## MATERIALS AND METHODS

### Data sources

The scRNA-seq data for the GSE98638 (n=6), GSE140228 (n=5), and GSE166635 (n=2) cohorts were downloaded from the Tumor Immune Single-Cell Hub 2 (TISCH 2) platform (http://tisch.comp-genomics.org/) [18]. Transcriptome matrices of the TCGA-LIHC cohort were obtained from the TCGA (https://portal.gdc.cancer.gov/repository) repository, which contains transcriptome data for 374 HCC tumour samples and 50 normal samples. Perl programming language was utilised for the transformation of simple nucleotide variation (SNV) data to further obtain the tumor mutation burden (TMB) values for each case in the TCGA-LIHC cohort. Transcriptomic and clinical information for GSE14520 (n=221) cohort was downloaded from the GEO (https://www.ncbi.nlm.nih.gov/) database, and case inclusion criteria were the presence of both transcriptomic and survival information. Clinical information and transcriptomic data for the CHCC validation cohort (n=159) were obtained from previous studies and the National Omics Data Encyclopedia database (https://www.biosino.org/node) [19]. Additionally, the data of ICGC-LIRI (n=232) were downloaded from the International Cancer Genome Consortium Data (ICGC; https://dcc.icgc.org/) portal. The Human Protein Atlas (HPA) was performed to obtain immunohistochemical staining images [20] (V.22.0, https://www.proteinatlas.org) (Supplementary Table 1). Tregs-related genes (TRGs) were downloaded from the Genecards (https://www.genecards.org/) (Supplementary Table 2) [21].

### Identification of differentially expressed genes (DEGs) between Tregs and other cells

R software (version R 4.1.2) was utilized to process scRNA-seq data (GSE98638, GSE140228 and GSE166635) to obtain DEGs between Tregs and other cells in each cohort (**|**fold change (FC)**|** > 1.5, False discovery rate (FDR) < 0.05). DEGs from the three cohorts were combined and defined as Tregs-related DEGs for subsequent analysis. The package “VennDiagram” was utilized to further plot Venn diagrams of Tregs-associated DEGs and TRGs to obtain differentially expressed TRGs (DETGs) for subsequent analysis.

### Identification of differentially expressed GETGs between HCC tumor and normal tissues

The R package “limma” was utilised to obtain differentially expressed GETGs (**|**FC**|** > 1.5, FDR<0.05) between tumor and normal tissues. Subsequently, the “pheatmap” was employed to draw differential expression heatmaps and volcano maps. The “sva” package was employed to eliminate batch effects and obtain expression data of differentially expressed GETGs in the TCGA and the GSE14520 cohorts.

### Construction of a TRSSys for HCC

Cox regression was used to obtain prognosis-associated GETGs in the TCGA-LIHC cohort, a process performed by the packages “survival” and “survminer”. Subsequently, the LASSO regression was used to screen the optimal TRGs for the establishment of TRSSys. The risk formula for TRSSys is as follows:


$$\text{TRSSys}=\sum_{\text{i}=1}^{\text{n}}\text{Coef}(\text{i})\text{Exp}(\text{i})$$

where Coef represents the regression coefficient of each TRGs in TRSSys. According to the regression coefficients, a higher TRSSys score represents a worse prognosis. Based on the TRSSys formula, the risk scores were calculated for each individual in the TCGA and the GEO testing cohorts, and all individuals were risk stratified according to the median risk score of the TCGA cohort.

### Validation and evaluation of TRSSys

First, the Kaplan-Meier (K-M) curves were utilised to determine the effect of the expression of TRSSys-related genes on the survival of individuals with HCC. The procedure was performed through the “survivor” and “survminer” packages. Additionally, the K-M method was also utilised to analyze the survival differences between patients in the high- and low-risk subgroups in the TCGA and GEO cohorts. Then, the “pheatmap” was performed to map the expression status of TRSSys-related genes. Furthermore, the K-M method was also used to assess the validity of TRSSys in the CHCC and ICGC-LIRI validation cohorts.

To further assess the effectiveness of TRSSys, we further conducted univariate (uni) and multivariate (multi) Cox analyses of TRSSys and selected clinicopathologic parameters to identify independent prognostic factors in the TCGA and GEO cohorts. Additionally, the ROC curves were employed to further assess the predictive efficacy of TRSSys in HCC. Finally, to assess the stability of TRSSys, we used the K-M method to determine whether TRSSys could discriminate between populations with different prognoses in different clinical subgroups.

### Development of TRSSys-based nomogram

TRSSys and tumor stage are independent prognostic factors for HCC. We developed a nomogram based on TRSSys and tumor stage for better determining the survival of individuals with HCC. The process was constructed utilizing the “regplot”, “survival” and “rms”. Moreover, Hosmer-Lemeshow calibration curves were utilised to assess whether the expected and actual probabilities calculated from the nomogram fit.

### TRSSys-based enrichment analysis

To analyze the differences in TRSSys-based risk stratification in pathway enrichment, we performed Gene Set Variation Analysis (GSVA) [22]. The “GSVA”, “GSEABase”, “pheatmap”, “ggplot2”, “reshape2” and “limma” were used to perform GSVA and to map the enrichment heatmap in the two risk subgroups. Further, the correlation between the expression of TRSSys-associated TRGs and different signaling pathways was also analyzed. To further explore the molecular differences between different risk subgroups, the “limma” was performed to obtain DEGs between the two risk subgroups (FC >2, FDR < 0.05). Gene Ontology (GO) and KEGG (Kyoto Encyclopedia of Genes and Genomes) were further utilised to analyze the enrichment of DEGs in functions and pathways and to visualize the outcome.

### TRSSys-based tumour mutation burden (TMB) analysis

To analyze the differences in mutation frequencies of genes in the different risk subgroups, the “maftools” package was utilised to map the waterfall of mutations in different subgroups. Additionally, “limma” was used to compare TMB between the two risk subgroups. Furthermore, the patients were categorized into four subgroups by two-by-two combinations of TMB subgroups and risk subgroups in the TCGA cohort, and the K-M curves were used to determine the differences in survival among the four subgroups of patients.

### TRSSys-based TIME analysis

TIMER is a platform for comprehensive assessment of immune infiltrates across different cancer types [23, 24]. To determine the correlation between TRSSys and various immune cell infiltrations, we acquired the immune cell data matrix of the TCGA cohort on the TIMER repository (http://timer.comp-genomics.org/) and performed Spearman’s analysis. The packages “scales”, “tidyverse”, “ggtext”, “ggpubr” and “ggplot2” were utilised to plot Spearman correlation bubble plots. To compare TIME differences between the two risk subgroups, we first performed ssGSEA on the TCGA-LIHC cohort to quantify the degree of immune cell infiltration in each sample, which in turn determines the corresponding immune-related function scores and immune-related cell scores. R packages “ggpubr” and “reshape2” were utilised to visualize differences in ssGSEA between risk subgroups. Activation of the immune checkpoint pathway was done as a key mechanism of tumor immune evasion [25]. We likewise analyzed the expression of major ICs in high- and low-risk subgroups and visualized the outcomes.

### TRSSys-based drug sensitivity analysis

OncoPredict is an algorithm that predicts drug sensitivity levels based on transcriptome expression levels [26]. The “limma”, “oncoPredict” and “parallel” packages were utilised to obtain a matrix of drug sensitivity data for the TCGA-LIHC cohort. Subsequently, the half-maximal inhibitory concentrations (IC50) of various agents in the two risk subgroups were analyzed to evaluate the value of TRSSys in guiding clinically individualized treatment.

### Statistical analysis

The statistical analyses involved in this study were done through R software (Vision 4.2.2) and its corresponding R packages. Survival analyses were performed using the K-M method. *P*-value < 0.05 indicates statistical significance.

## RESULTS

### Identification of Tregs in HCC

**Figure 1:**
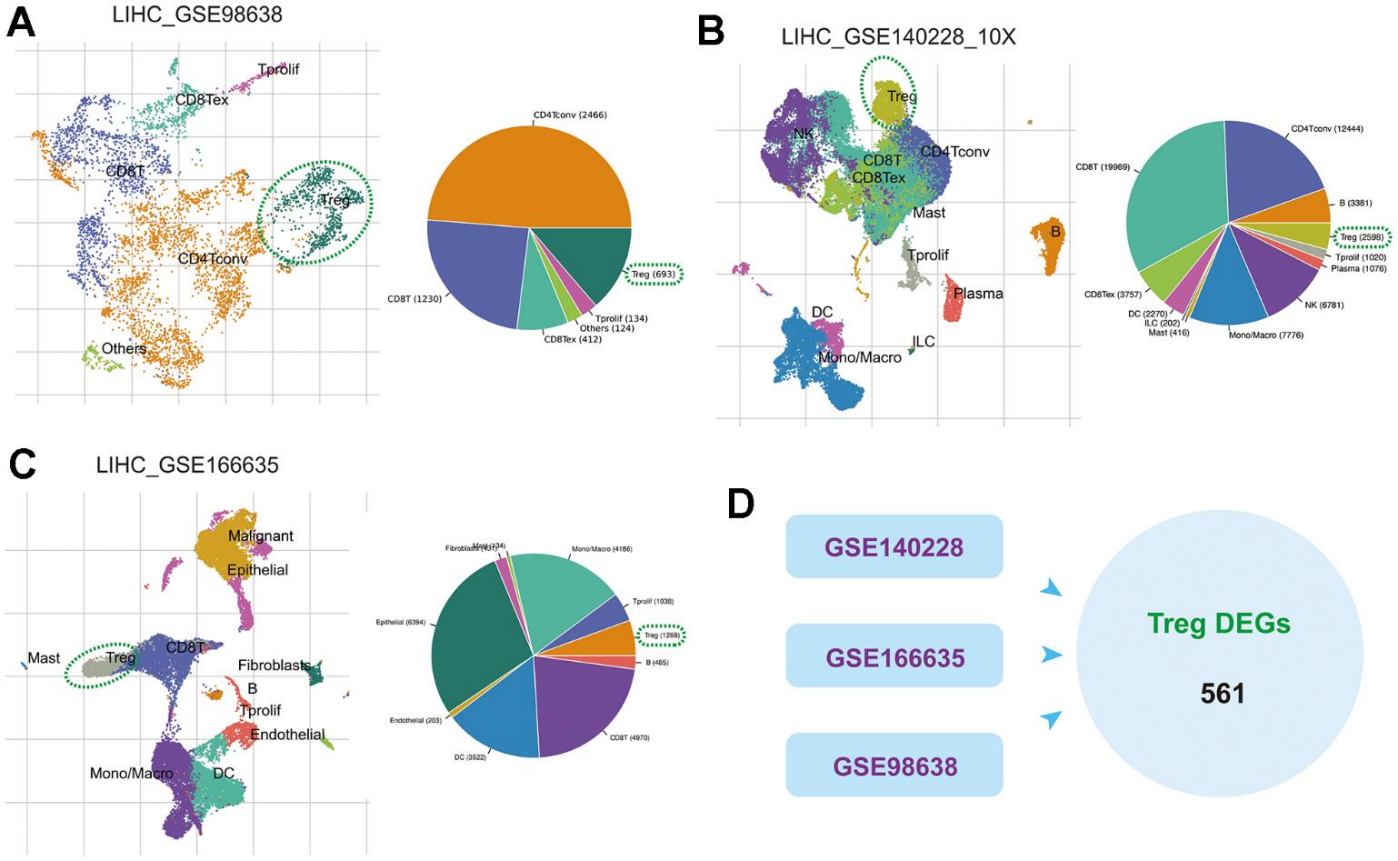
**The scRNA-seq analysis identifies Tregs in HCC.** (**A**–**C**) Annotation of cell clusters in the GSE98638, GSE140228 and GSE166635 cohorts. (**D**) DEGs between Tregs and other cell types in these three cohorts.

Tregs of the GSE98638, GSE140228 and GSE166635 cohorts were identified and annotated according to the TISCH cell marker annotations (Figure 1A–1C). Subsequently, differential analysis extracted DEGs between Tregs and other cell types in these three HCC cohorts and merged them to obtain 561 Tregs-associated DEGs for subsequent analysis (Figure 1D).

### Identification of DETGs in HCC

**Figure 2:**
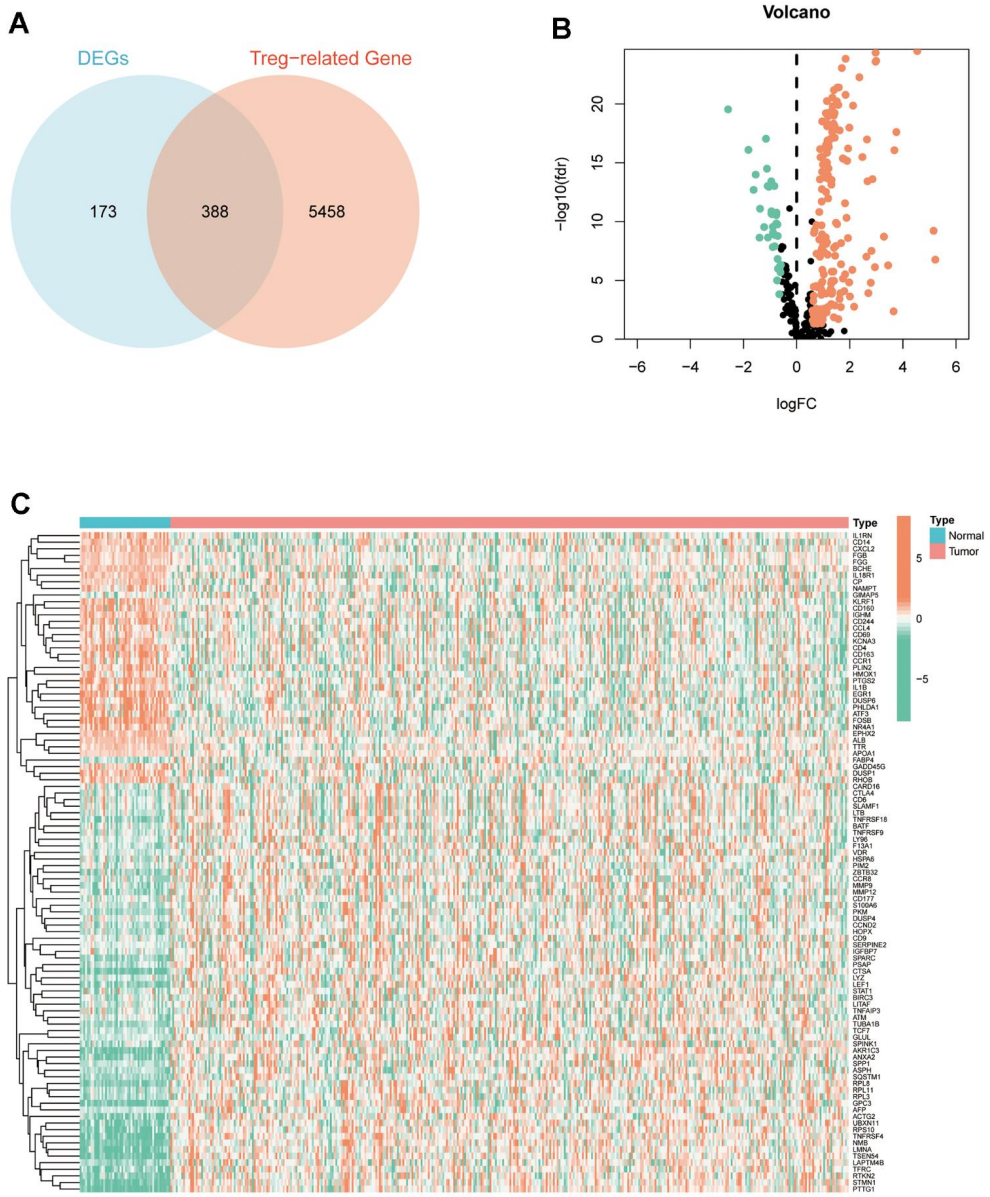
**Identification of DETGs.** (**A**) Intersected 5846 TRGs with the 561 DEGs to obtain 388 DETGs. (**B**) The volcano plot demonstrated 388 differentially expressed DETGs in HCC tumour and normal tissues. (**C**) The heatmap demonstrated the expression of 100 differentially expressed DETGs in HCC tumour and normal tissues.

We obtained 5846 TRGs on Genecards (relevance score >10), and intersected these TRGs with the 561 DEGs above to obtain 388 DETGs for subsequent analysis (Figure 2A). The volcano plot demonstrated 388 differentially expressed DETGs in HCC tumour tissues and normal tissues, which contained 36 DETGs that were lowly expressed in tumour tissues and 171 DETGs that were highly expressed in tumour tissues (Figure 2B). Additionally, the heatmap demonstrated the expression of 100 differentially expressed DETGs in HCC tumour and normal tissues (Figure 2C).

### Identification of TRSSys in HCC

**Figure 3:**
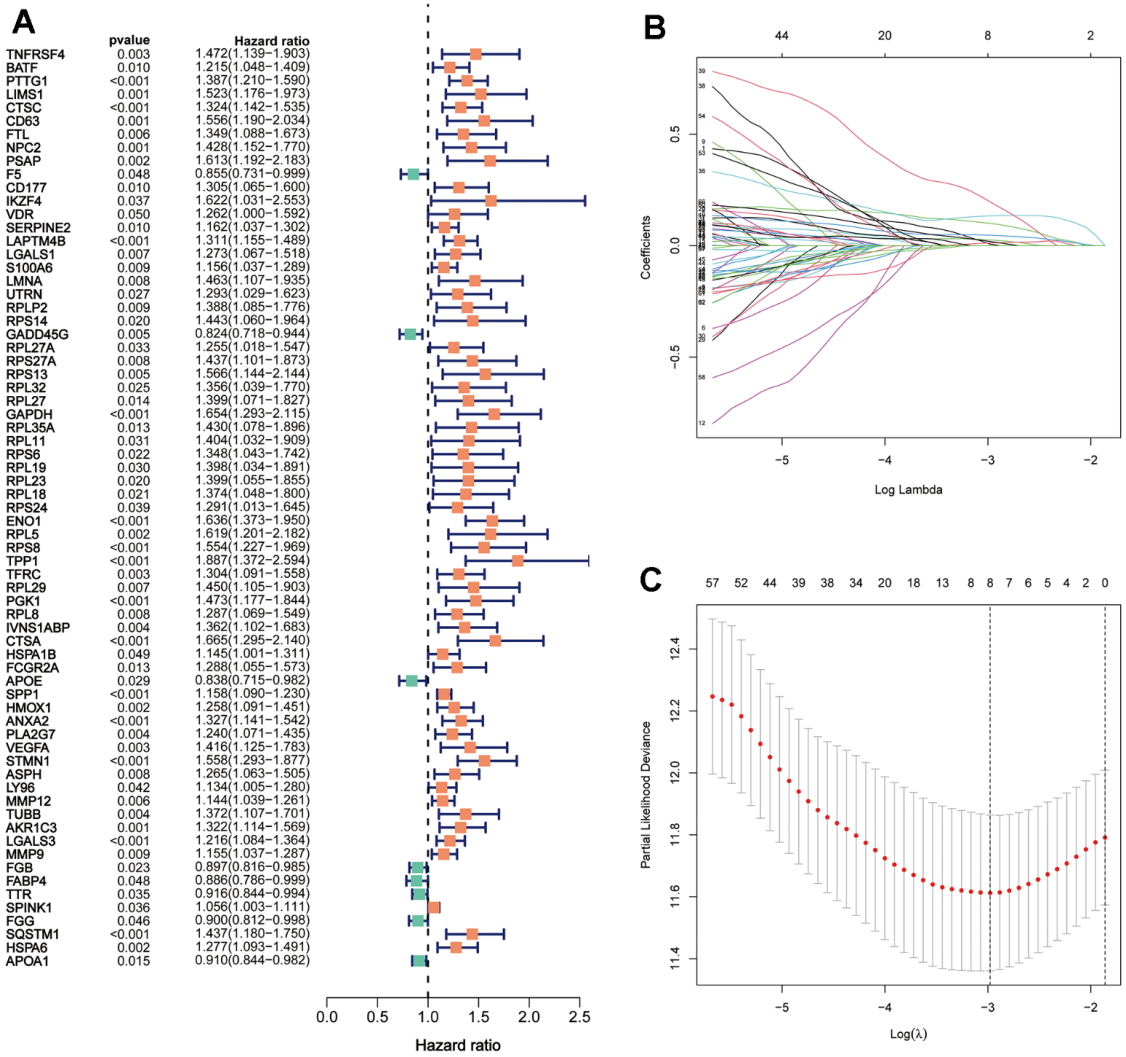
**Establishment of TRSSys for HCC.** (**A**) The 69 TRGs associated with HCC prognosis. (**B**, **C**) The variable selection and cross-validation plots based on the LASSO.

**Figure 4:**
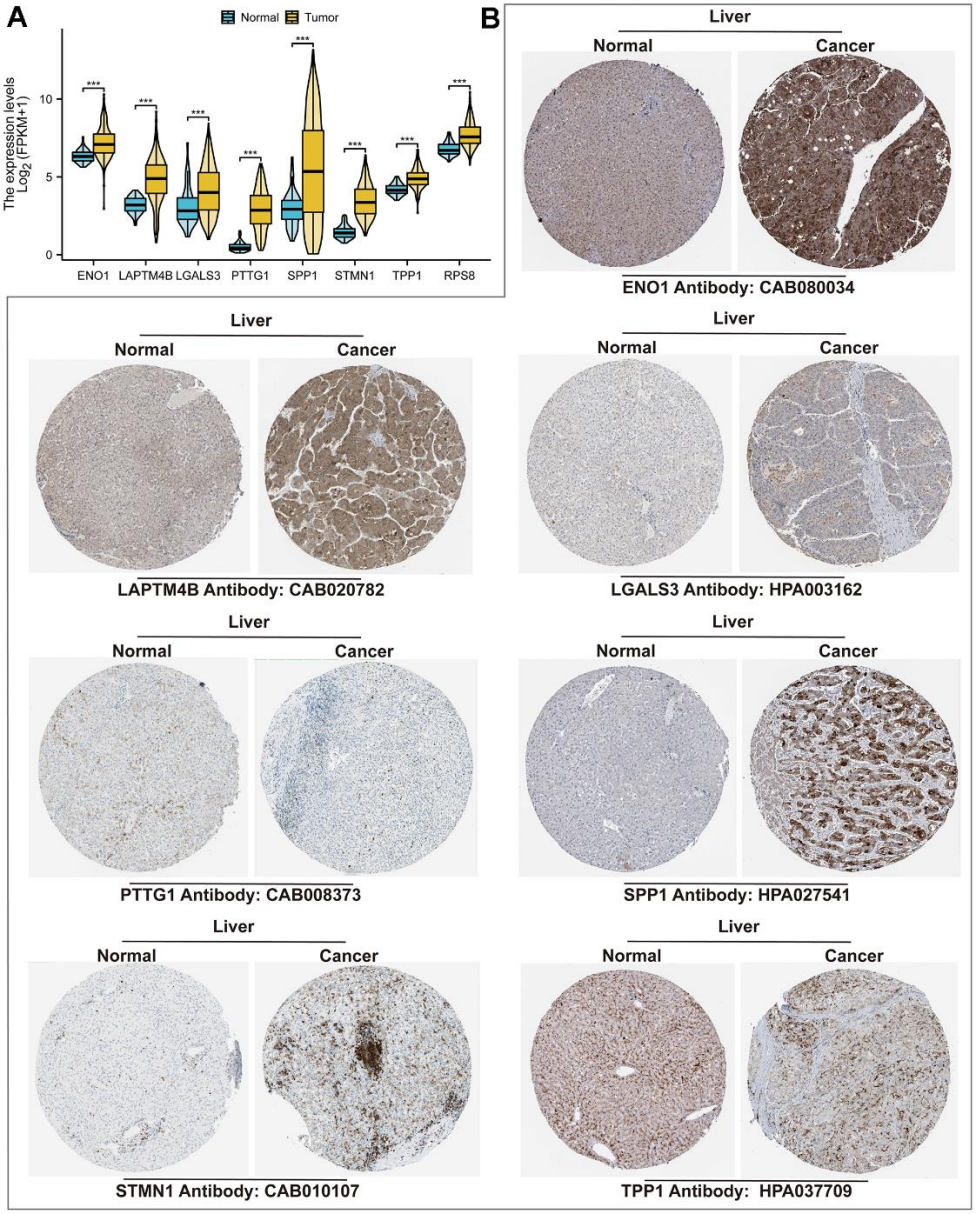
**TRSSys-related genes in HCC.** (**A**) Differential expression of TRSSys-related TRGs in tumor and normal tissues. (**B**) Immunohistochemical images of TRSSys-related TRGs in the HPA.

**Figure 5:**
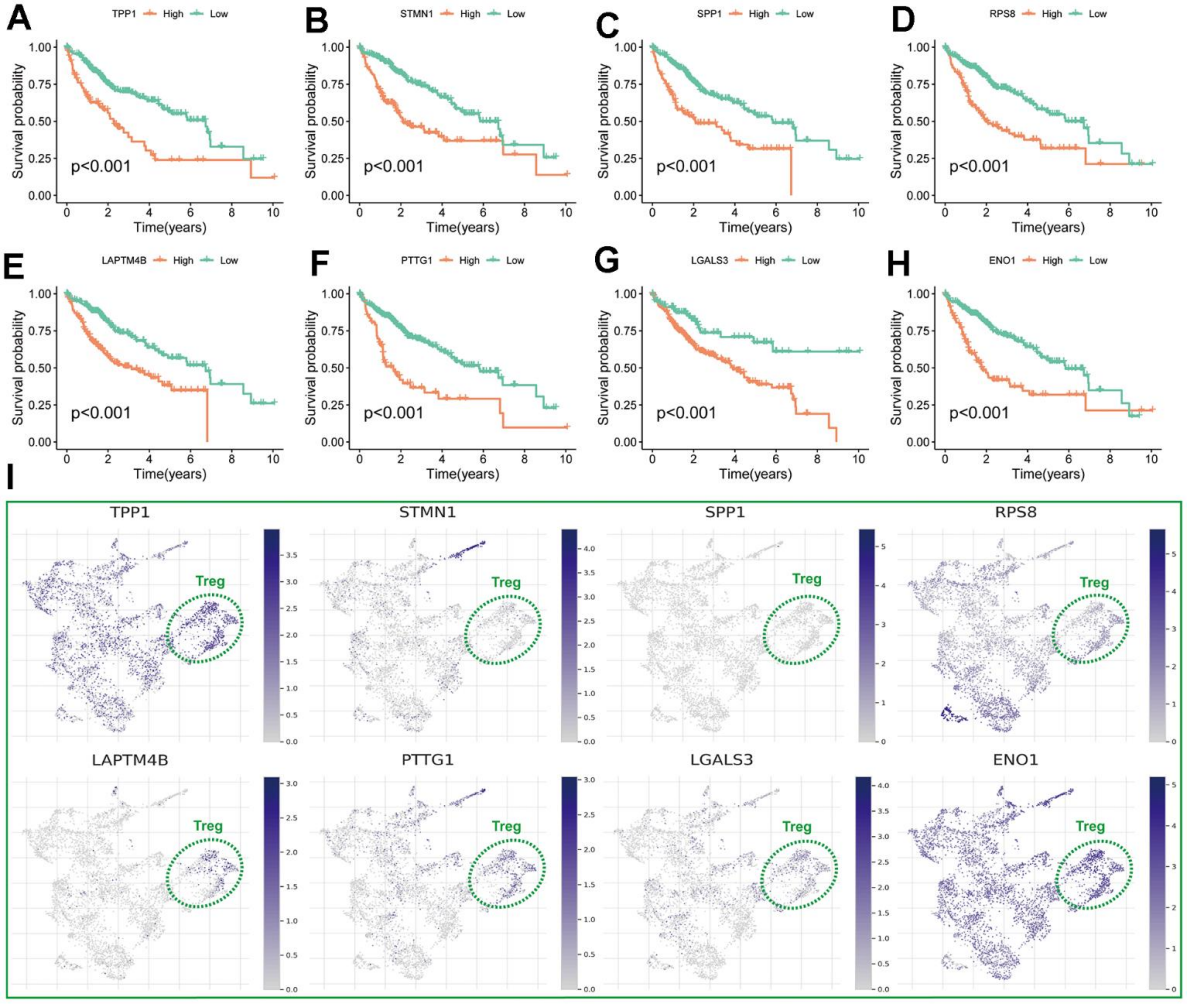
**Assessment of TRSSys-related genes in HCC.** (**A**–**H**) K-M curves of the 8 TRSSys-related for OS in the TCGA cohort. (**I**) The expression of TRSSys-associated TRGs in Tregs of GSE98638 cohort.

Cox regression was performed on the differentially expressed DETGs to identify 69 TRGs associated with HCC prognosis (Figure 3A). LASSO regression finally screened 8 of these TRGs for TRSSys construction (Figure 3C, 3D) (Table 1). All of these TRGs were highly expressed in HCC tumour tissues (Figure 4A, 4B). In addition, the K-M curves revealed that the high expression of all these 8 TRGs was a poor prognostic factor (Figure 5A–5H). Furthermore, Figure 5I demonstrated the expression of TRSSys-associated TRGs in Tregs of GSE98638 cohort. Subsequently, according to the risk formula for TRSSys, the risk score = Exp (PTTG1) * (0.10034) + Exp (LAPTM4B) * (0.02591) + Exp (ENO1) * (0.13373) + Exp (RPS8) * (0.00719) + Exp (TPP1) * (0.19610) + Exp (SPP1) * (0.04681) + Exp (STMN1) * (0.01250) + Exp (LGALS3) * (0.01890). The risk score for each individual was calculated from the above equation.

**Table 1:** Treg-associated scoring system.

**Gene**	**Coef**	**HR**	**HR.95L**	**HR.95H**	***p*-value**
PTTG1	0.10034	1.38733	1.21038	1.59015	< 0.001
LAPTM4B	0.02591	1.31142	1.15503	1.48898	< 0.001
ENO1	0.13373	1.63610	1.37292	1.94972	< 0.001
RPS8	0.00719	1.55416	1.22662	1.96917	< 0.001
TPP1	0.19610	1.88674	1.37227	2.59409	< 0.001
SPP1	0.04681	1.15773	1.08954	1.23019	< 0.001
STMN1	0.01250	1.55761	1.29281	1.87664	< 0.001
LGALS3	0.01890	1.21621	1.08423	1.36427	< 0.001

HR, hazard ratio; Coef, coefficient.

### Verification of TRSSys in HCC

**Figure 6:**
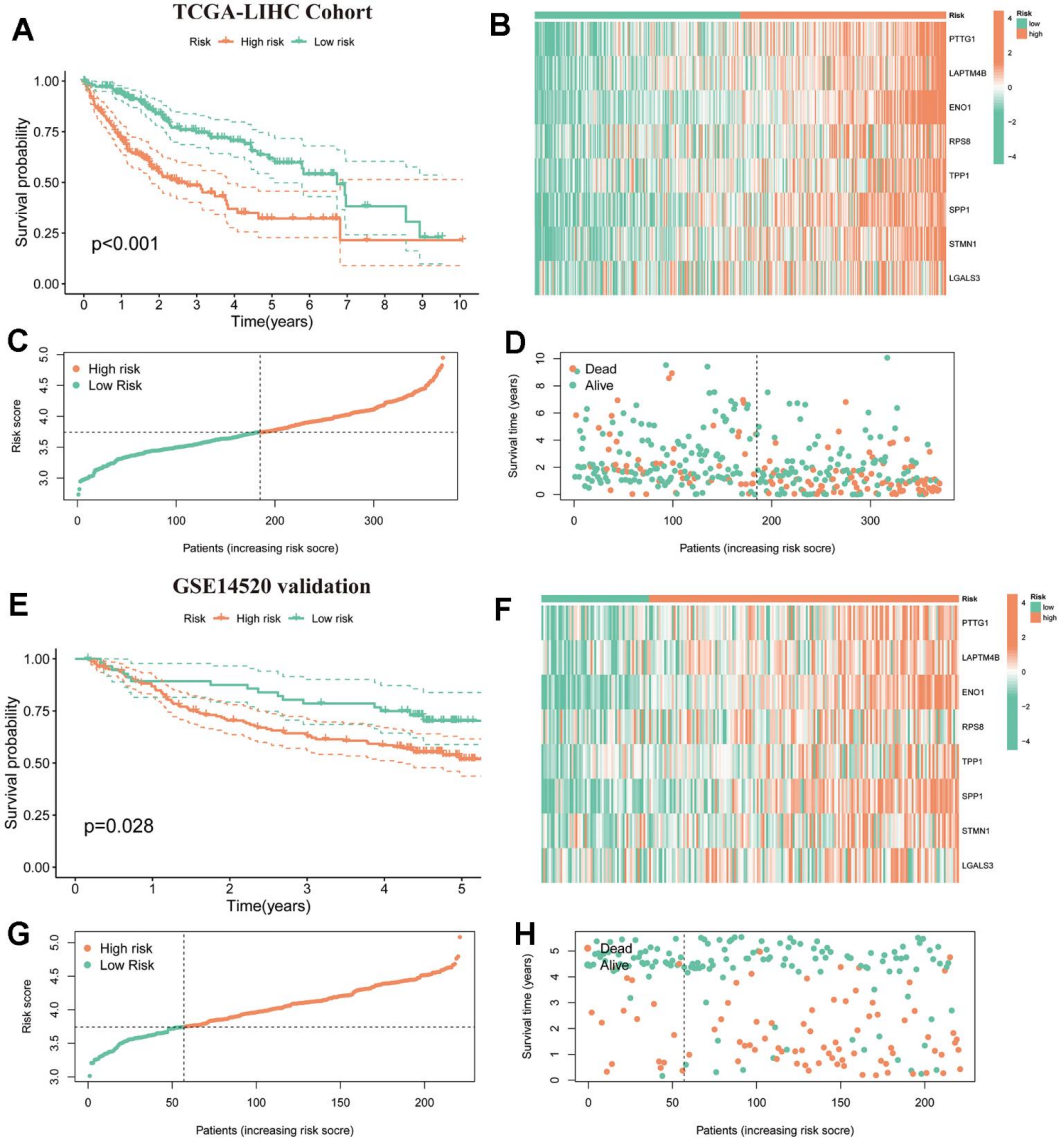
**Verification of TRSSys in HCC.** (**A**) Kaplan-Meier curves in the TCGA-LIHC. (**B**) Expression heat map of TRSSys-related TRGs in the TCGA cohort. (**C**, **D**) The distribution of survival conditions in the TCGA-LIHC. (**E**) Kaplan-Meier curves in the GSE14520. (**F**) Expression heat map of TRSSys-related TRGs in the GSE14520 cohort. (**G**, **H**) The distribution of survival conditions in the GSE14520.

We first assessed the predictive ability of TRSSys in the TCGA cohort. K-M curves (Figure 6A) suggested that the survival of low-risk patients was superior to that of the high one (*P*<0.001). The expression heat map suggested that TRSSys-related TRGs were expressed at higher levels in the high-risk group (Figure 6B). Risk curves and risk status plots suggested that the number of patients with mortality increased as the risk score increased (Figure 6C, 6D). To further validate TRSSys, we analyzed the predictive value of TRSSys in an independent validation cohort, GSE14520. The K-M curves also suggested that the survival of low-risk patients was superior to that of the high one (*P*=0.028) (Figure 6E). The expression heat map also suggested that TRSSys-associated TRGs had higher expression levels in the high-risk group (Figure 6F). Risk curves and risk status plots suggested that the proportion of patients with mortality status increased as the risk score increased (Figure 6G, 6H). Moreover, the K-M curves of the CHCC (Supplementary Figure 1A) and ICGC-LIRI (Supplementary Figure 1B) cohorts further validated the effectiveness of TRSSys in HCC.

### Evaluation of the TRSSys in HCC

**Figure 7:**
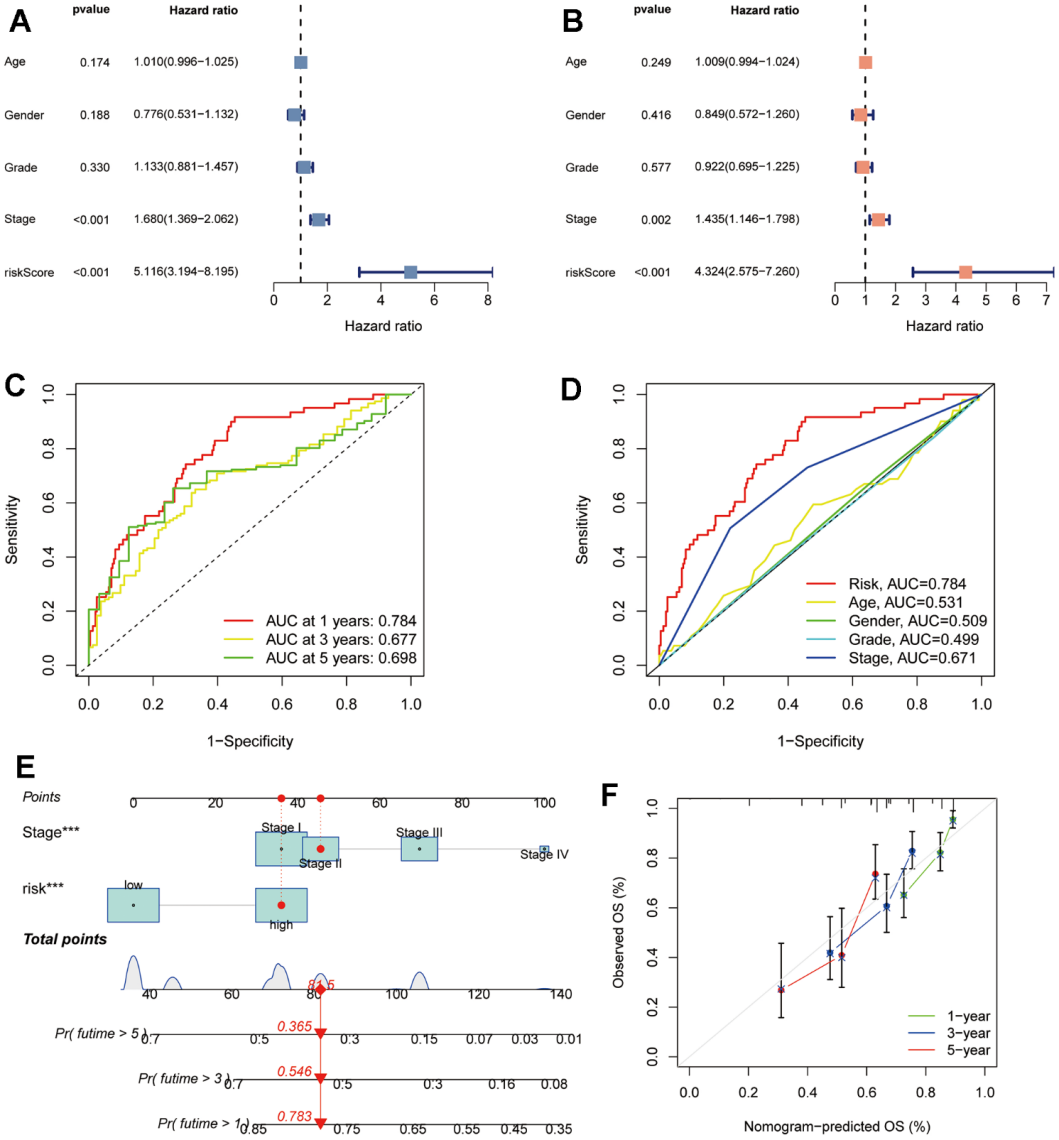
**Assessment of TRSSys in HCC.** (**A**, **B**) Forest plots for univariate (**A**) and multivariate Cox (**B**) regression analysis in the TCGA cohort. (**C**) ROC curves for the TRSSys in the TCGA cohort. (**D**) Comparison of the TRSSys with clinicopathological parameters in the TCGA cohort. (**E**) Nomogram for predicting OS in HCC. (**F**) Calibration curves for nomogram. *P < 0.05, **P < 0.01, and ***P < 0.001.

Both uni- and multi-Cox regression suggested that TRSSys could independently predict the clinical outcomes of individuals with HCC, with hazard ratios of 5.116 and 4.324 (*P* < 0.001) (Figure 7A, 7B). Furthermore, TNM stage was another independent prognostic indicator. Moreover, the ROC curves showed that the AUC values of TRSSys in predicting 1-, 3-, and 5-year OS were 0.784, 0.677, and 0.698 (Figure 7C). Comparisons of AUC values of TRSSys with age, gender, grading, and staging in the ROC curves also suggested the good predictive efficacy of TRSSys (Figure 7D). Additionally, in the GSE14520 cohort, Cox regression also showed that TRSSys was an independent prognostic factor in patients with HCC (Supplementary Figure 2A, 2B). The ROC curves in the GSE14520 showed that the AUC values of TRSSys predicting 1-, 3- and 5-year OS were 0.596, 0.624 and 0.662, respectively (Supplementary Figure 2C, 2D).

### TRSSys-based nomogram

Cox regression indicated that both TRSSys and stage were prognostic variables for HCC. To better predict the survival of clinical individuals, we incorporated TRSSys-based risk stratification and stage into the construction of the nomogram (Figure 7E). The 1-, 3-, and 5-year OS rates for a stage II and high-risk individual were estimated to be 0.783, 0.546, and 0.365, respectively, based on the nomogram. The calibration curves revealed that the expected probabilities and the actual probabilities were in high agreement (Figure 7F).

### TRSSys-based clinical parameter stratification

**Figure 8:**
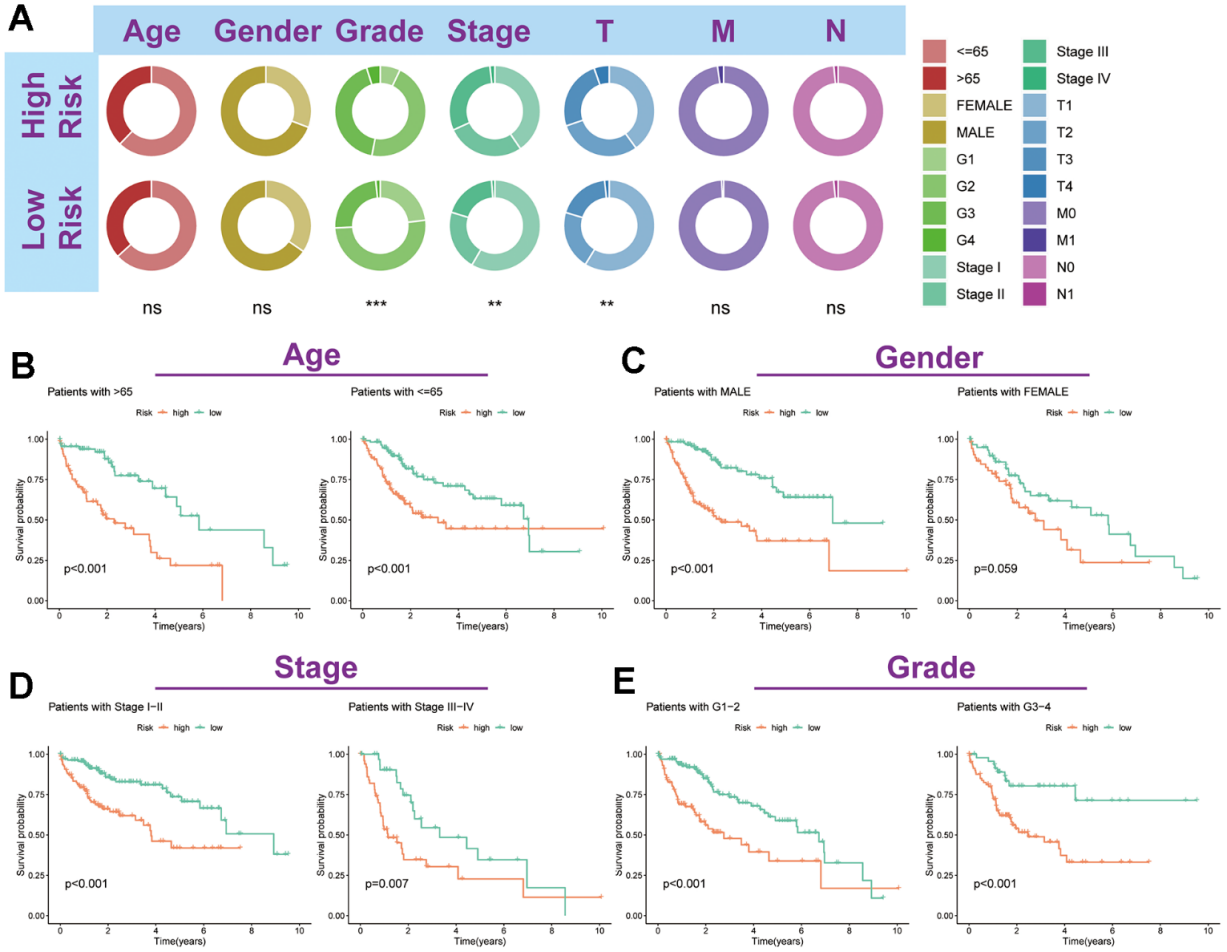
**Association of TRSSys with clinical parameters in HCC.** (**A**) Distribution status of different clinicopathologic parameters in two risk subgroups. (**B**–**E**) Kaplan-Meier curves revealed the survival between individuals in the two risk groups for age (**B**), gender (**C**), stage (**D**) and grade (**E**) subgroups.

Circle plots demonstrate the status of clinicopathological parameters in the two risk groups (Figure 8A). Furthermore, K-M curves showed that patients with different age, stage and grade had a worse prognosis in the high-risk group (Figure 8B–8E). Although there was no significant difference in survival among females in the two risk subgroups (*P* = 0.059), a trend toward separate survival curves could still be seen. Together, these results suggest the general applicability of TRSSys in HCC.

### GSVA, GO and KEGG

**Figure 9:**
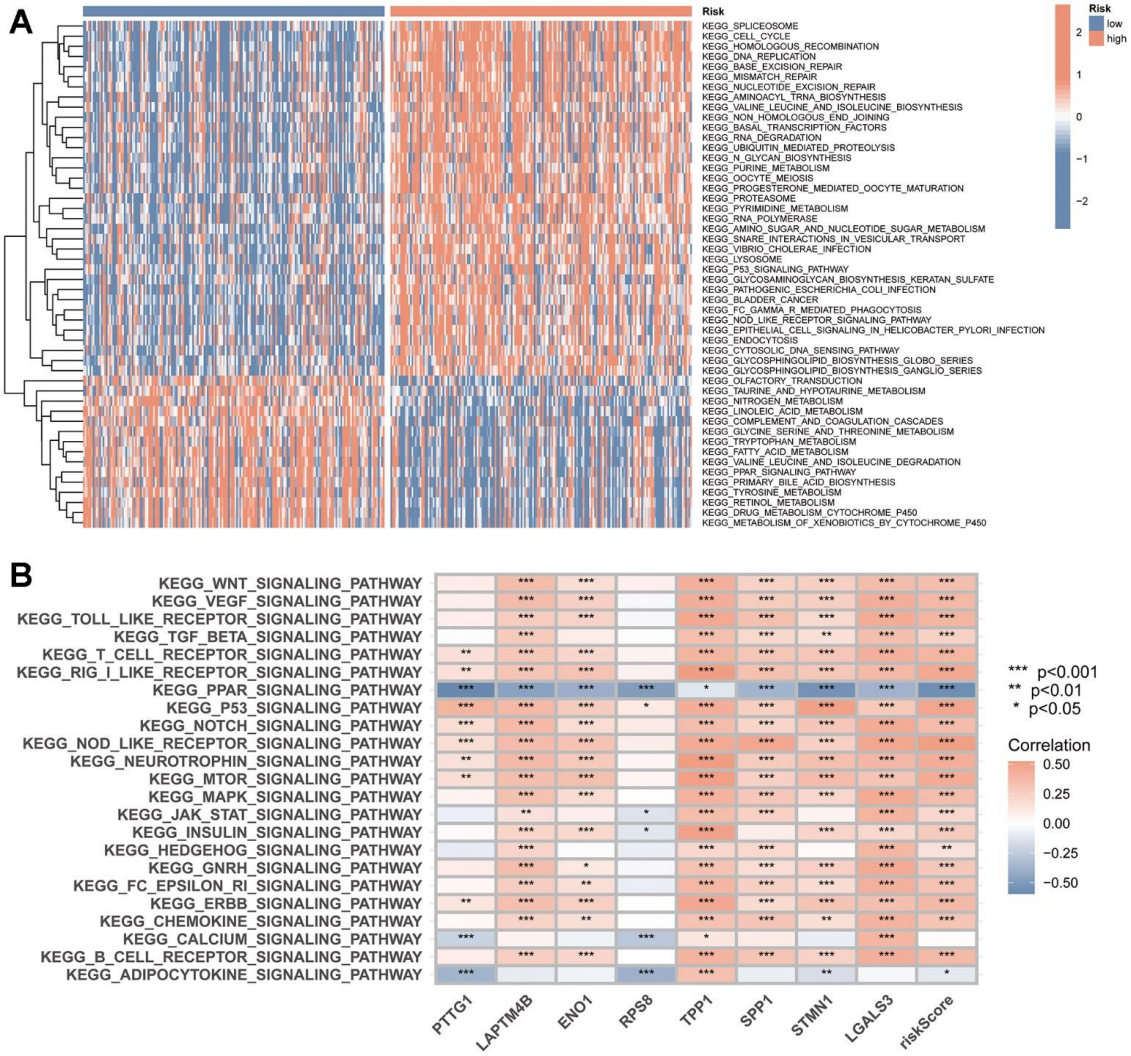
**TRSSys-based GSVA.** (**A**) KEGG enriched in the high- and low-risk groups. (**B**) The correlation between the expression of TRSSys-related TRGs and tumour-related pathways.

GSVA results showed that the functions enriched in the high-risk subgroup included cell cycle, homologous recombination, DNA replication, basic excision repair, mismatch repair, nucleotide excision repair, purine metabolism, RNA polymerase, RNA degradation, lysosome, P53 signalling pathway and NOD-like receptor signalling pathway (Figure 9A). Additionally, correlation heatmaps showed that the expression of TRSSys-associated TRGs was closely related to the signalling pathway (Figure 9B).

**Figure 10:**
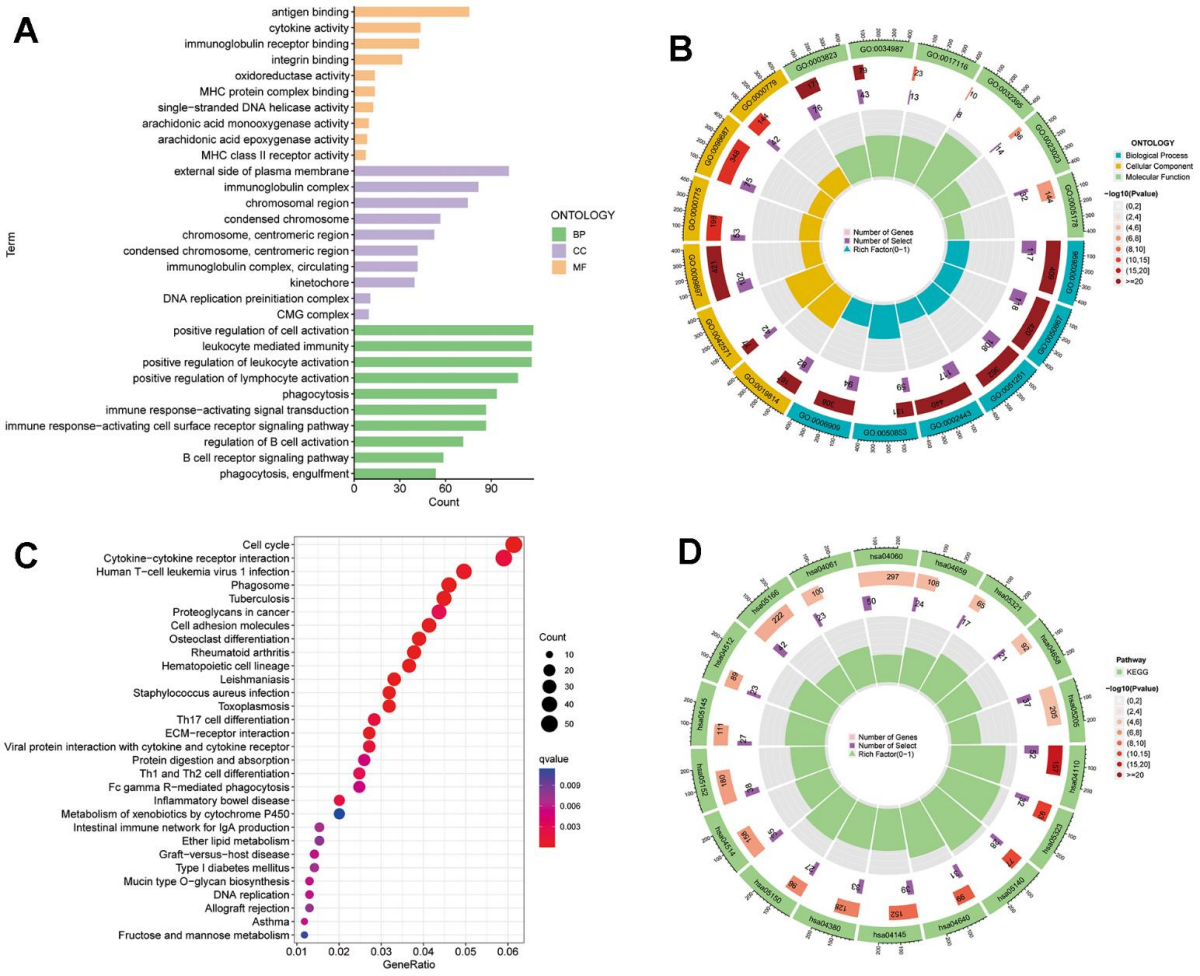
**TRSSys-based GO and KEGG analysis.** (**A**, **B**) GO analysis of the DEGs between the risk subgroups. (**C**, **D**) KEGG analysis of the DEGs between the risk subgroups.

DEGs were identified between the high- and low-risk subgroups and further explored the enrichment of DEGs in biological functions. GO analysis revealed that in terms of biological processes, DEGs were mainly enriched in positive regulation of cell activation, leukocyte mediated immunity, positive regulation of leukocyte and lymphocyte activation, phagocytosis and immune response-activating signal transduction. Regarding cellular component, DEGs are mainly enriched in external side of plasma membrane, immunoglobulin complex, chromosomal region, and condensed chromosomes. Regarding molecular function, DEGs are enriched in functions such as antigen binding, cycline activity, immunoglobulin receptor binding, and integrin binding (Figure 10A, 10B). Finally, KEGG analysis showed that DEGs were enriched in cell cycle, cyclokine-cyclokine receptor interaction, phagosome, and proteoglycans in cancer (Figure 10C, 10D).

### TRSSys-based TMB analysis

The mutation waterfall plot showed the mutational differences in the 20 genes with the highest mutation frequencies in HCC across risk groups (Supplementary Figure 3A, 3B). Additionally, TMB levels were not significantly different in the high- and low-risk groups (Supplementary Figure 3C). However, there was a difference in survival between TMB subgroups and risk subgroup combinations, with the best prognosis for low-TMB and low-risk combinations (*P* < 0.001) (Supplementary Figure 3D). The above results suggest that the combination of TRSSys and TMB in HCC may be able to better determine the prognosis of patients.

### TRSSys predicts TIME in HCC

**Figure 11:**
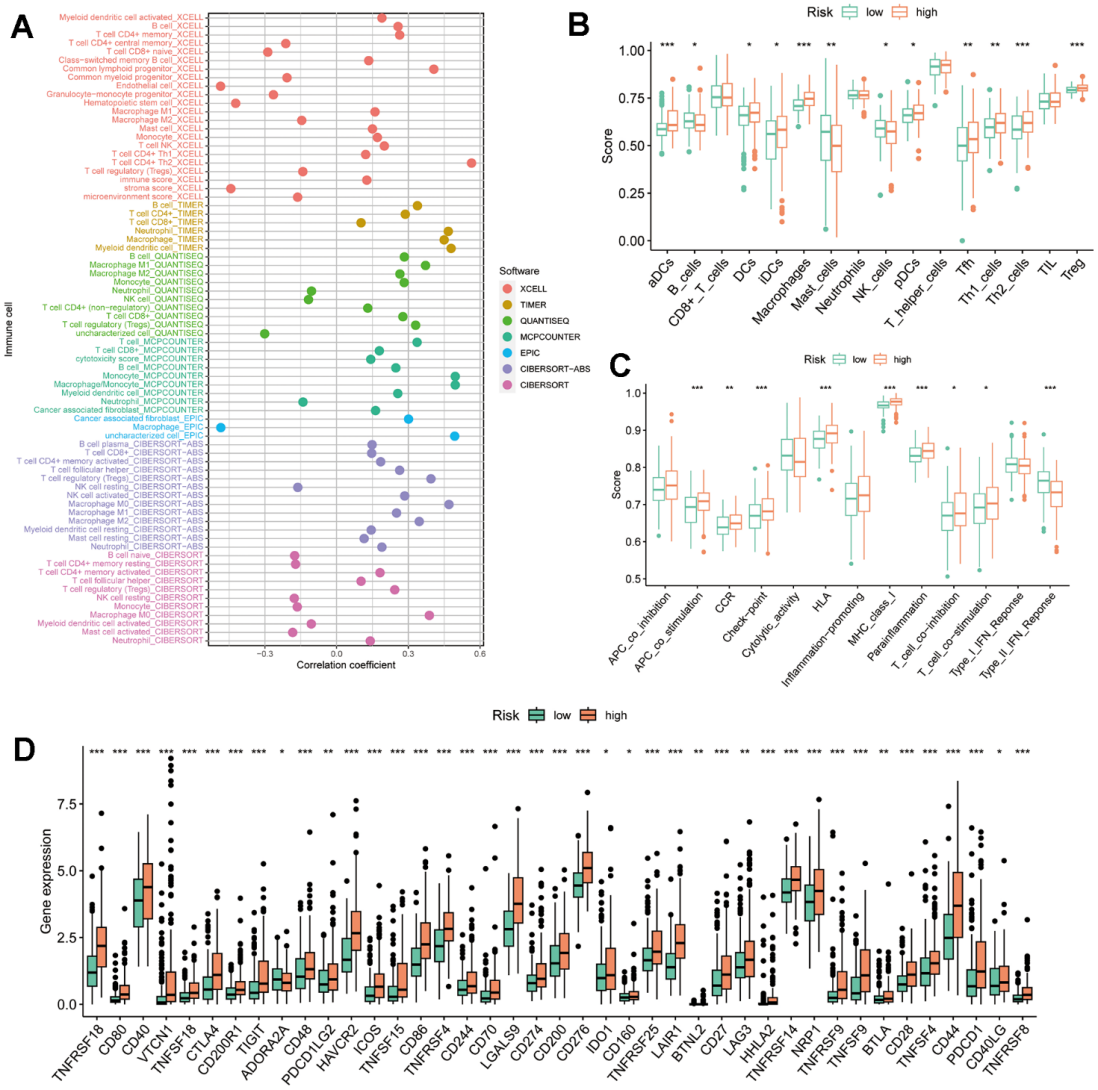
**TRSSys-based TIME analysis.** (**A**) Correlation bubble plot between the degree of immune cell infiltration and risk score. (**B**, **C**) Differences in immune cell scores and immune function scores across risk subgroups. (**D**) Differences in expression of immune checkpoints across risk subgroups.

The correlation between TRSSys and TIME of HCC was further analyzed considering the regulatory role of Tregs on TIME. The immune correlation bubble plots suggested a positive correlation between risk score and immune score in the XCELL algorithm; a positive correlation between risk score and CD4+ T cells, CD8+ T cells, macrophages, and neutrophils in the TIMER algorithm; and a positive correlation between macrophages, Treg cells, and immune score in QUANTISEQ. In addition, most immune cells were positively correlated with the risk score in the EPIC, and CIBERSORT-ABS algorithms (Figure 11A). The ssGSEA results revealed that patients in the high-risk group had significantly higher levels of infiltration of activated dendritic cells, dendritic cells, macrophages, follicular helper T cells, helper T cells and Tregs (Figure 11B). Notably, in terms of immune function, immune checkpoints also had higher expression levels in the high-risk subgroup (Figure 11C). Further immune checkpoint-associated gene analysis revealed that most checkpoints were significantly elevated in the high-risk group (Figure 11D), suggesting a highly immunosuppressive state in the high-risk group.

### TRSSys predicts drug sensitivity in HCC

**Figure 12:**
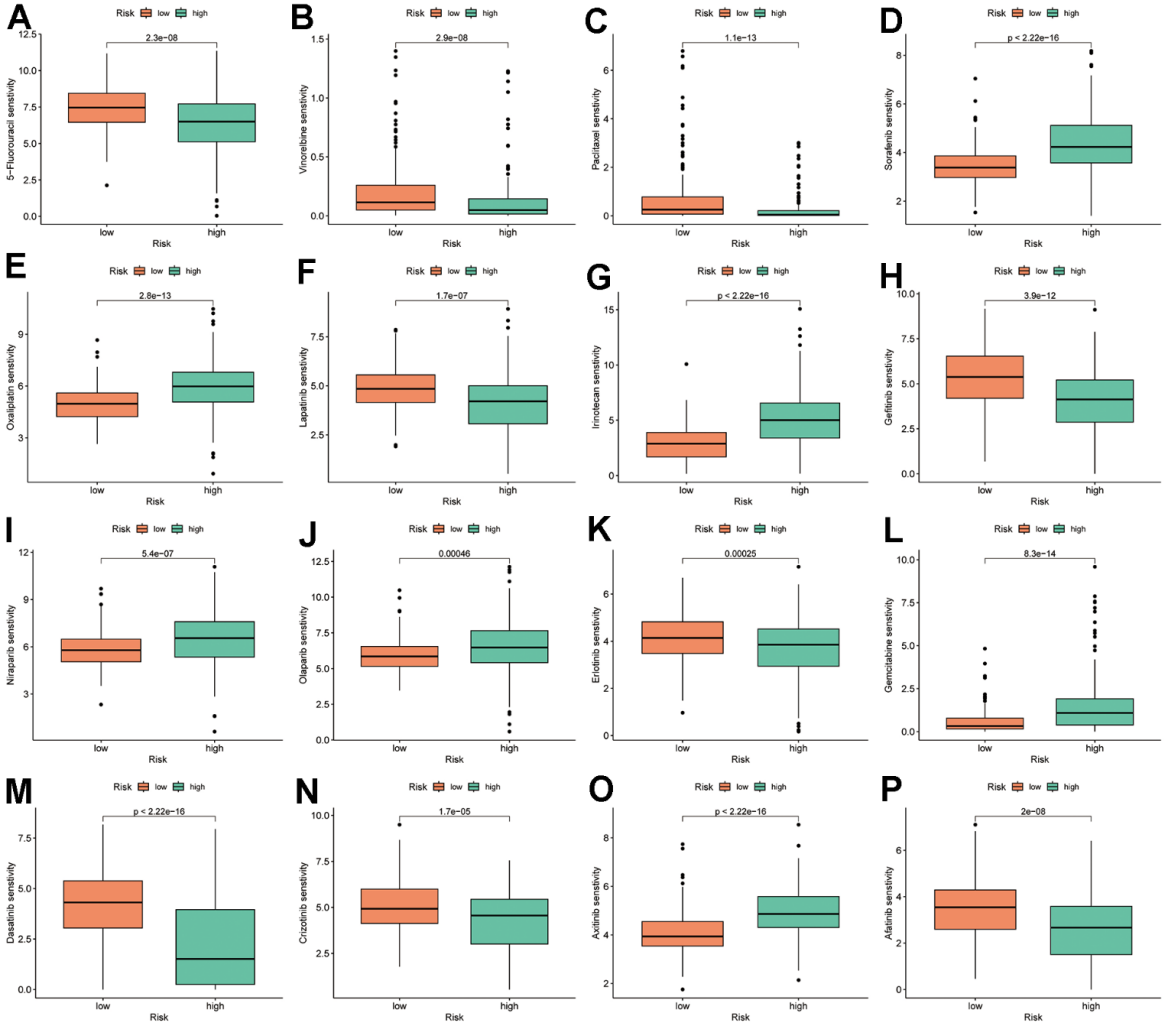
**TRSSys-based IC50 analysis.** (**A**–**P**) Therapy response of common chemotherapy and targeted drugs for risk groups.

TRSSys-based drug sensitivity analysis revealed that the IC50 of multiple chemotherapeutic and targeted drugs differed between risk subgroups (*P* < 0.001) (Figure 12A–12P). Among them, 5-fluorouracil, vinorelbine, paclitaxel, lapatinib, gefitinib, erlotinib, dasatinib, crizotinib, and afatinib showed significantly lower IC50 in the low-risk group. Additionally, sorafenib, oxaliplatin, irinotecan, niraparib, olaparib, gemcitabine, and axitinib had significantly higher IC50s in the low-risk group.

## DISCUSSION

Malignant tumours are currently one of the major causes of death worldwide and have become a major category of diseases that seriously jeopardize human life and health and constrain socio-economic development. The occurrence and development of malignant tumours not only depend on the continuous growth signals and unlimited replication and proliferation capabilities possessed by the tumor cells themselves, but are also closely related to the inhibitory TIME shaped by the tumour cells, which allows them to evade the surveillance and killing by the body’s immune system [27]. Tregs are important immune-negative regulatory cells in TIME, which reduce sensitivity to antigen recognition by affecting antigen-presenting cells and dendritic cells [28]. Additionally, Tregs persistently express glucocorticoid-induced tumour necrosis factor receptor family-associated protein (GITR) and cytotoxic T-lymphocyte-associated protein 4 (CTLA-4), which inhibit effector T-cell activity [29, 30] and bind to IL-2 with high affinity, depleting IL-2 in the microenvironment and impeding the maintenance of function and maturation of Tconv cells [31]. Furthermore, elevated Treg ratios in tumour-infiltrating immune cells (TICs) have been shown to correlate with a poorer prognosis in some tumours [32, 33]. Moreover, tumor-infiltrating Tregs are considered key targets for cancer immunotherapy, either as monotherapy and/or in combination with ICIs antibodies [15]. Therefore, it is of great importance to explore the potential value of Tregs in assessing prognosis, TIME and immunotherapy efficacy in individuals with cancer.

In the current study, a novel TRSSys was developed based on Tregs for the first time to predict prognosis in individuals with HCC. The results revealed that TRSSys-based risk stratification could well distinguish patients with different prognoses and was an independent prognostic factor for HCC. The ROC curves confirmed the superior predictive power of TRSSys over clinicopathologic parameters alone. Stratified analysis of clinical parameters suggested broad applicability of TRSSys. Furthermore, the prognostic predictive value of TRSSys was further validated in the independent validation cohort GSE17538. Together, these results suggest that TRSSys has favorable prognostic predictive efficacy in HCC.

Among the TRSSys, TPP1 was the TRGs with the highest regression coefficients. Previous studies have shown that TPP1 is associated with macrophages in HCC [34], suggesting that there may be a crosstalk of TPP1 between different immune cells. ENO1, which is second only to TPP1 in terms of coefficient in the scoring system, has been shown to promote hepatocarcinogenesis through YAP1-dependent arachidonic acid metabolism and has been implicated in HCC in relation to oxidative stress [35, 36]. Additionally, PTTG1 can promote HCC evolution through reprogramming of asparagine metabolism and is a potential therapeutic and diagnostic target for HCC [37]. LAPTM4B as a risk factor in a scoring system which has been shown to induce autophagy and promote tumour growth in HCC [38]. LGALS3 has been shown to be used as a potential biomarker for the malignant progression of HBV infection to HCC and is thought to be associated with necroptosis in HCC [39]. Notably, the role of RPS8 in HCC has not been elucidated, and given the prognostic predictive value of TRSSys for HCC, its regulatory mechanism in HCC warrants further subsequent exploration.

Over the past decade, immunotherapy has become an important therapeutic tool in antitumor therapy after surgery, chemotherapy, radiotherapy and targeted therapy, bringing new hope to patients with cancer [40, 41]. In 2011, ipilimumab became the first Food and Drug Administration (FDA)-approved ICB therapy targeting the immune checkpoint CTLA-4 [42]. Since then, a variety of ICB drugs targeting immune checkpoint programmed cell death 1 (PD-1) and its ligand PD-L1 have been approved [43, 44]. Currently, ICB agents as monotherapy or in combination with chemotherapy or targeted therapies have become the standard of care for a wide range of tumors, including metastatic melanoma, lung, kidney, and liver [40, 45]. Nevertheless, the clinical application of ICB drugs still faces the bottleneck problem of low overall efficacy. Therefore, it is meaningful to explore reliable efficacy prediction biomarkers to identify the beneficiary population of ICB therapy.

There is growing evidence that ‘immune hot tumours’ are more effective for ICB immunotherapy [46, 47]. One of the most important features of ‘immune hot tumours’ is the activation of immune checkpoints including PD-1, CTLA-4, and lymphocyte activation gene 3 (LAG3) [46]. In this study, checkpoints PD-1, LAG3, PD-L1 and CTLA-4 showed lower levels of expression in the low-risk population identified based on TRSSys, indicating a high degree of suppression of TIME in high-risk populations, which partly explains the poorer prognosis of the high-risk population. Additionally, the results of ssGSEA and immune checkpoint analysis together suggest that the high-risk group is more consistent with ‘immune hot tumours’ and may be a potential beneficiary population for ICB immunotherapy.

Treatment strategies for HCC are limited by the underlying condition of the patient. Despite the rapid development of surgical and localized treatments in recent years, 50% to 60% of patients still require systemic therapy [48]. Currently, the commonly used systemic therapeutic drugs in the clinic, in addition to the ICB drugs mentioned above, are the targeted therapeutic drugs represented by tyrosine kinase inhibitors (TKIs). Based on SHARP and ORIENTAL results, sorafenib was approved for the first time for unresectable HCC, with a 2.8-month prolongation of median survival time in the targeted therapy group compared to the placebo group [49]. The results of TRSSys-based IC50 analysis revealed that the low-risk subgroup was more sensitive to sorafenib. Currently, resistance to TKIs remains one of the challenges in the treatment of advanced HCC. The Poly adenosine diphosphate ribose polymerase (PARP) inhibitor olaparib is thought to overcome sorafenib resistance by remodeling the pluripotent transcriptome in HCC [50]. Additionally, another PARP inhibitor, niraparib, was shown to induce HCC cytotoxicity along with significant autophagy formation and autophagic flux [51]. In the present study, patients in the low-risk subgroup were similarly more sensitive to olaparib and niraparib, indicating that the high-risk subgroup may be resistant to PARP inhibitors.

The present study evaluated the constructed scoring system by Cox, ROC, and other methods and validated it with an independent external cohort, but there are still some limitations. First, we currently lack data from our own large sample of hepatocellular carcinoma samples and prospective studies to validate the scoring system. Moreover, the mechanism of the regulatory effects of scoring system-related TRGs on Tregs in HCC deserves to be further explored in the future.

## CONCLUSIONS

In the present study, a novel TRSSys was developed for the first time based on Tregs in HCC, which can efficiently predict clinical outcomes in patients with HCC. Furthermore, risk stratification based on TRSSys can identify populations with highly suppressive TIME and assist in determining potentially advantageous populations for ICB immunotherapy. Finally, TRSSys can also provide a basis for clinical individualized treatment decisions for patients with advanced HCC.

## Supplementary Material

Supplementary Figures

Supplementary Table 1

Supplementary Table 2
