# Supplementary material for: Comprehensive assessment of regulatory T-cells-related scoring system for predicting the prognosis, immune microenvironment and therapeutic response in hepatocellular carcinoma
Source: Aging (Albany NY). 2024 Mar 8;16(6):5288–310. doi: 10.18632/aging.205649 (PMC11006487; doi:10.18632/aging.205649)
Supplement: Supplementary Figures [file aging-16-205649-s001.pdf]

SUPPLEMENTARY FIGURES

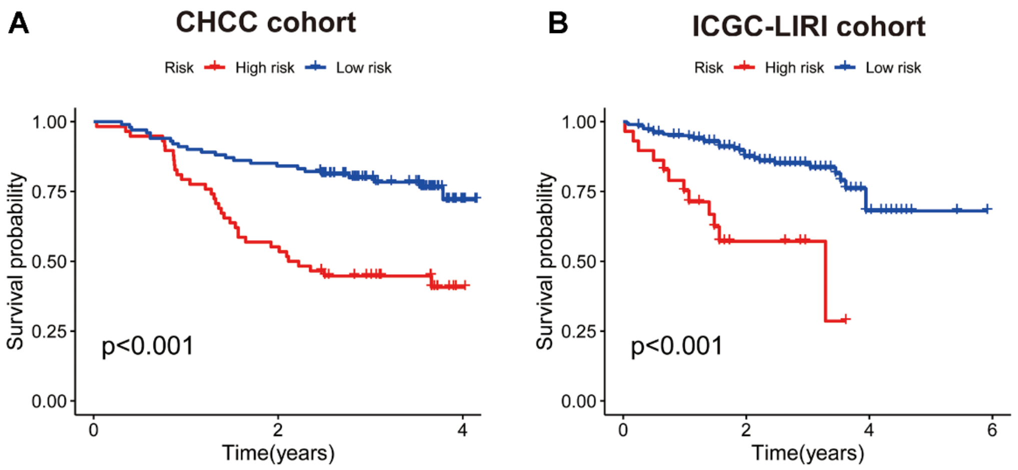

Supplementary Figure 1. K-M curves for the validation cohorts. (**A**) K-M curves for the CHCC cohort. (**B**) K-M curves for the ICGC-LIRI cohort.

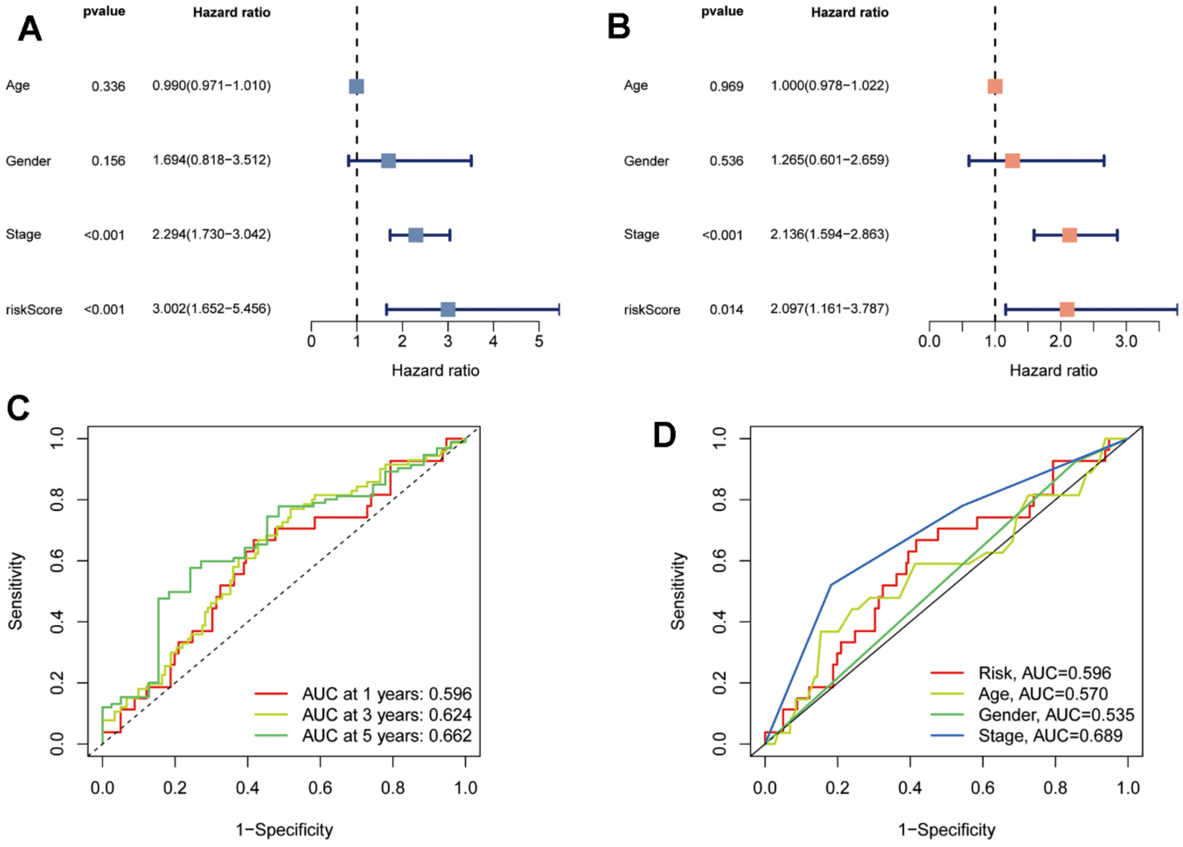

Supplementary Figure 2. Assessment of TRSSys in HCC. (**A**, **B**) Forest plots for univariate (**A**) and multivariate Cox (**B**) regression analysis in the GSE14520 cohort. (**C**) ROC curves for the TRSSys in the GSE14520. (**D**) Comparison of the TRSSys with clinicopathological parameters in the GSE14520.

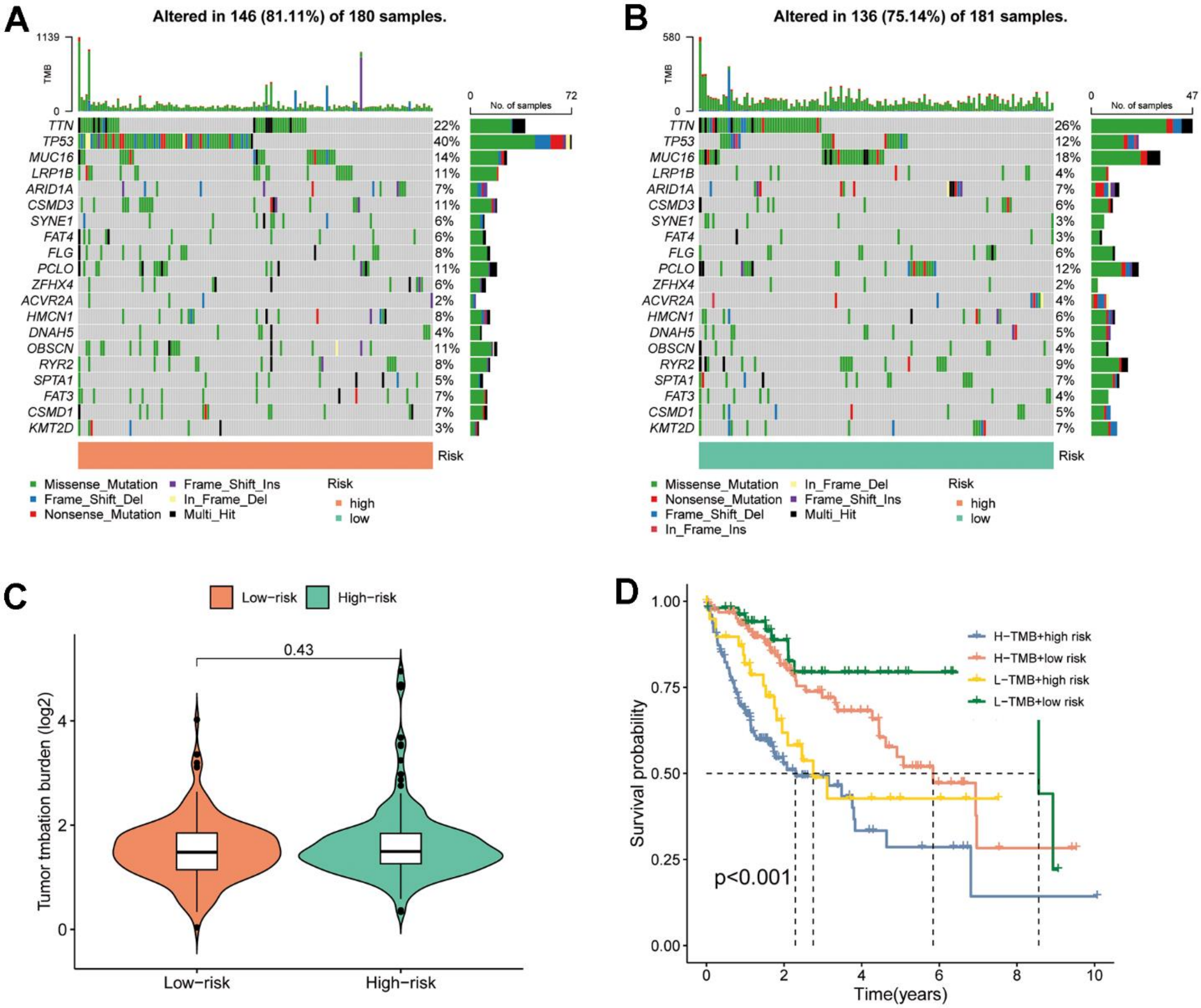

Supplementary Figure 3. TRSSys-based TMB analysis. (**A**, **B**) The waterfall plots showing mutation information in each tumor sample of different risk groups. (**C**) TMB level between two risk groups. (**D**) Kaplan-Meier curves based on the combination of TMB and risk status.
