# Supplementary material for: Comprehensive assessment of regulatory T-cells-related scoring system for predicting the prognosis, immune microenvironment and therapeutic response in hepatocellular carcinoma
Source: Aging (Albany NY). 2024 Mar 8;16(6):5288–310. doi: 10.18632/aging.205649 (PMC11006487; doi:10.18632/aging.205649)
Supplement: Supplementary Table 1 [file aging-16-205649-s002.pdf]

SUPPLEMENTARY TABLE

Supplementary Table 1. Immunohistochemical staining images of TRSSys-related genes in HPA (URL link).

TRSSys-related genes	Human protein atlas (URL link)
ENO1	https://www.proteinatlas.org/ENSG00000074800-ENO1/tissue/liver<br>https://www.proteinatlas.org/ENSG00000074800-ENO1/pathology/liver+cancer
LAPTM4B	https://www.proteinatlas.org/ENSG00000104341-LAPTM4B/tissue/liver<br>https://www.proteinatlas.org/ENSG00000104341-LAPTM4B/pathology/liver+cancer
LGALS3	https://www.proteinatlas.org/ENSG00000131981-LGALS3/tissue/liver<br>https://www.proteinatlas.org/ENSG00000131981-LGALS3/pathology/liver+cancer
PTTG1	https://www.proteinatlas.org/ENSG00000164611-PTTG1/tissue/liver<br>https://www.proteinatlas.org/ENSG00000164611-PTTG1/pathology/liver+cancer
SPP1	https://www.proteinatlas.org/ENSG00000118785-SPP1/tissue/liver<br>https://www.proteinatlas.org/ENSG00000118785-SPP1/pathology/liver+cancer
STMN1	https://www.proteinatlas.org/ENSG00000117632-STMN1/tissue/liver<br>https://www.proteinatlas.org/ENSG00000117632-STMN1/pathology/liver+cancer
TPP1	https://www.proteinatlas.org/ENSG00000166340-TPP1/tissue/liver<br>https://www.proteinatlas.org/ENSG00000166340-TPP1/pathology/liver+cancer
